# Alternative Functional *rad21* Paralogs in *Fusarium oxysporum*

**DOI:** 10.3389/fmicb.2019.01370

**Published:** 2019-06-18

**Authors:** Manish Pareek, Yael Almog, Vinay Kumar Bari, Einat Hazkani-Covo, Itay Onn, Shay Covo

**Affiliations:** ^1^Department of Plant Pathology and Microbiology, The Robert H. Smith Faculty of Agriculture, Food and Environment, The Hebrew University of Jerusalem, Rehovot, Israel; ^2^Department of Natural Sciences, Open University of Israel, Ra’anana, Israel; ^3^The Azrieli Faculty of Medicine, Bar Ilan University, Safed, Israel

**Keywords:** cohesin, Mcd1, Rad21, Rec8, *Fusarium oxysporum*, cell cycle

## Abstract

Cohesin, the sister chromatid cohesion complex, is an essential complex that ensures faithful sister chromatid segregation in eukaryotes. It also participates in DNA repair, transcription and maintenance of chromosome structure. Mitotic cohesin is composed of Smc1, Smc3, Scc3, and Rad21/Mcd1. The meiotic cohesin complex contains Rec8, a Rad21 paralog and not Rad21 itself. Very little is known about sister chromatid cohesion in fungal plant pathogens. *Fusarium oxysporum* is an important fungal plant pathogen without known sexual life cycle. Here, we describe that *F. oxysporum* encodes for three Rad21 paralogs; Rad21, Rec8, and the first alternative Rad21 paralog in the phylum of ascomycete. This last paralog is found only in several fungal plant pathogens from the Fusarium family and thus termed *rad21*nc (non-conserved). Conserved *rad21* (*rad21*c), *rad21*nc, and *rec8* genes are expressed in *F. oxysporum* although the expression of *rad21*c is much higher than the other paralogs. *F. oxysporum* strains deleted for the *rad21*nc or *rec8* genes were analyzed for their role in fungal life cycle. Δ*rad21*nc and Δ*rec8* single mutants were proficient in sporulation, conidia germination, hyphal growth and pathogenicity under optimal growth conditions. Interestingly, Δ*rad21*nc and Δrec8 single mutants germinate less effectively than wild type (WT) strains under DNA replication and mitosis stresses. We provide here the first genetic analysis of alternative *rad21*nc and *rec8* paralogs in filamentous fungi. Our results suggest that *rad21*nc and *rec8* may have a unique role in cell cycle related functions of *F. oxysporum*.

## Introduction

*Fusarium oxysporum* is a soil-borne plant pathogen that infects more than hundred plant species and causes severe yield losses (Dean et al., 2012). *F. oxysporum* has polyphyletic origin with lineage-specific chromosomes that encode for pathogenicity genes (Ma et al., 2010, 2013). These lineage specific chromosomes are mobile; they can be transferred between isolates passaging pathogenic traits (Ma et al., 2010). In addition, *F. oxysporum* is capable of exchanging segments of chromosomes between isolates although a sexual life cycle was never identified. This suggests that parasexual recombination does occur in this fungus (Vlaardingerbroek et al., 2016). Very little is known about chromosome transmission and parasexual recombination at the mechanistic level in *F. oxysporum*. The motivation of this study is to identify *F. oxysporum*-specific chromosomal proteins. The long term goal is to assess the role of these proteins in chromosome transactions that may specifically occur in *F. oxysporum*. A small scale comparative genomic study led us to focus on the cohesin complex.

The cohesin complex consists of Smc1, Smc3, Rad21 (also known as Mcd1 or Scc1), and Scc3 (Onn et al., 2008; Nasmyth and Haering, 2009). In the meiotic cohesin complex, the Rad21 subunit is replaced by its meiosis specific paralog Rec8 (Bhatt et al., 1999; Watanabe and Nurse, 1999). From an evolutionary standpoint the cohesin complex is part of a broad family of proteins found in prokaryotes and eukaryotes containing the SMC motives. In the cohesin family of proteins; the kleisin subunit bridges between the two SMC subunits (Nasmyth and Haering, 2005; Gligoris et al., 2014; Palecek and Gruber, 2015). There are several kleisin families; here we focus on α-kleisins that are part of the cohesin complex (Nasmyth and Haering, 2005). The α-kleisins subunits connect Smc1 and Smc3 by binding their globular heads. In yeast, the Mcd1 (Rad21) N-terminal is bound to Smc3 and the C-terminal to Smc1 (Haering et al., 2002). Besides binding Smc1 and Smc3 in eukaryotes α-kleisins contain separase cleavage sites that allow destruction of the cohesin complex in anaphase (Uhlmann et al., 1999). The cohesin complex holds the newly replicated sister chromatids till anaphase thus it ensures the proper segregation of chromatids (Spencer et al., 1990; Guacci et al., 1997; Onn et al., 2008; Covo et al., 2012, 2014). Cohesin also has an important role in determining the efficiency and fidelity of homologous recombination by facilitating recombination between sister chromatids and excluding recombination between homologous chromosomes (Sjögren and Nasmyth, 2001; Ünal et al., 2004; Covo et al., 2010). During meiosis, an alternative form of cohesin is formed that functions in a different way; meiotic cohesins suppress recombination between sister chromatids and facilitate recombination between homologous chromosomes (Zickler and Kleckner, 1999; Kim et al., 2010).

Cohesin also functions in transcription regulation of mRNA and rRNA (Lengronne et al., 2004; Bose and Gerton, 2010). It has a major role in maintaining 3D chromatin structure by supporting chromatin loops formations that bring distal genome parts together (Kagey et al., 2010; Li et al., 2013; Kakui and Uhlmann, 2018; van Ruiten and Rowland, 2018 and the references therein).

All the subunits of cohesin are well conserved across eukaryotes, however, there are several examples of lineage-specific gene duplication of some subunits. In *Arabidopsis thaliana* two *RAD21* paralogs (*AtRAD21.2/SYN3* and *AtRAD21.3/SYN4*) have a role in somatic DNA double strand break repair (Dong et al., 2001; da Costa-Nunes et al., 2006, 2014; Bolaños-Villegas et al., 2017). In addition, *SYN1* encodes for a Rad21/Rec8 like protein that functions in meiosis. *Arabidopsis SYN1* gene mutants are male and female sterile, defective in chromosome condensation and pairing start at leptotene stage of meiosis I. *SYN1* is dispensable for somatic and vegetative growth though (Bai et al., 1999; da Costa-Nunes et al., 2014). *DIF1* is another *Arabidopsis* homolog of Rec8/Rad21, mutants are completely male and female sterile and showed multiple meiotic defects in *Arabidopsis* (Bhatt et al., 1999).

In *Caenorhabditis elegans* and mammals, it was shown that at least two *RAD21*/*REC8* paralogs function in a non-redundant manner in meiosis (Severson et al., 2009; Ishiguro et al., 2011; Severson and Meyer, 2014). In conclusion, all reported Rad21 paralogs were shown to be functional. To the best of our knowledge, only two Rad21 paralogs were reported in fungi, the mitotic Rad21 and the meiotic Rec8. Interestingly, even fungi without known sexual life cycle like *F. oxysporum* encode for *rec8*. Here, we report that three paralogs of *rad21* are encoded in the genome of *F. oxysporum*. One is the conserved, canonical *rad21* (*rad21*c), another is a non-conserved *rad21* (*rad21*nc) and a meiotic specific, i.e., *rec8*. Based on genetic analysis of *rad21*nc and *rec8* paralogs in *F. oxysporum* we suggest that the alternative paralogs are also functional and they may have a Fusarium-specific function in cell cycle regulation.

## Materials and Methods

### Phylogenetic Analysis of the *rad21* Paralogs

Protein sequences were collected from 12 fungal species for *rad21* paralogs and analyzed; the orthologous were aligned using MAFFT v7.221 (Katoh et al., 2009) with default parameters for protein alignment. Guidance2 was used to remove untrustable positions from the alignment with a Guidance score below score 0.93 (Sela et al., 2015). A phylogenetic tree with 100 bootstrap replicates was then reconstructed using RAxML v8.2.11 (Stamatakis, 2014) with GAMMA model of rate heterogeneity (Yang, 1994) and the GTR substitution model. Species tree was reconstructed by 90 DNA Repair proteins listed in Supplementary Table S1.

### Analysis of Rad21nc Sequence and Predicted Structures

Sequence alignments were done with ClustalX2 (Larkin et al., 2007). Structural predication was done by using I-TASSER (Zhang, 2008; Roy et al., 2010; Yang et al., 2015) PDB 1W1W and 4UX3 were assigned as modeling templets for *F. oxysporum* Rad21nc sequence (Haering et al., 2004; Gligoris et al., 2014).

### Fungal Strain and Culture Conditions

All experiments described here used *F. oxysporum* f. sp. *lycopersici* strain 4287 (Fungal Genetics Stock Center #9935). Glycerol stock of original fungal strain is maintained at -80°C. For spore isolation, spores were inoculated in 50 ml in KNO_3_ (1.36 gm yeast nitrogen base, 24 gm sucrose, 100 mM KNO_3_ in 800 mL distilled water) medium in Erlenmeyer flask and incubated in 28°C at 250 rpm for 4–6 days. The mycelia/spore suspension was then filtered using a cell strainer (40 μm, SPL Life Sciences, South Korea), the filtrates were centrifuged and washed twice with distilled water. Spores were diluted and counted using a Neubauer counting chamber. Spores and mycelia were also grown on potato dextrose agar (PDA) plates and incubated at 28°C for 5–7 days for various experiments.

### RT-PCR and qPCR Expression Analysis for *rad21* Paralogs

RT-PCR was performed to quantify the expression of the different *rad21* paralogs in WT and mutant strains. RNA was isolated from spores 8 h post inoculation using Plant RNAeasy kit (Qiagen, United States). Then, cDNA was made by FastQuant RT Kit (Tiangen Biotech, China). Further, *rad21* paralogs and *act1* (*actin*) genes were amplified using specific primes (P16 to P23) described in Supplementary Table S2. SYBR green (Thermo Fisher Scientific, United States) was used for qPCR analysis using StepOnePlus^TM^ Real-time PCR system (Applied Biosystems, United States).

To analyze the effect of cell cycle arrest on the expression of *rad21* paralogs fungal spores (0.2 billion) germinated in 10 mL of potato dextrose broth (PDB) with or without 100 mM hydroxyurea (HU) or 50 μg/mL benomyl for 14 h at 28°C, 250 rpm. RNA isolation and cDNA preparation was done as described above. *act1* gene served as an internal control. Fold change were calculated relative to PDB germinated spores using the ΔΔC_T_ method (Livak and Schmittgen, 2001).

### Generation of Δ*rad21*nc or Δ*rec8* Strains

The deletion constructs for *F. oxysporum rad21*nc or *rec8* genes were prepared using the split marker approach as previously described, with few modifications (Catlett et al., 2003; Yu et al., 2004). The strategy for preparation of the split cassette is described in Supplementary Figure S1. Briefly, 620 bp upstream flanking region and 672 bp long downstream region of *rad21*nc coding region (FOXG_15850) were amplified from *F. oxysporum* genomic DNA using primers P1/P2 and P3/P4, respectively (Supplementary Table S2). Hygromycin cassette (HYG) was amplified using primers P5/P6 from pSilent-1 vector (Fungal Genetics Stock Center, Manhattan, KS, United States) (Nakayashiki et al., 2005). Further combined region with upstream *rad21*nc and a half portion of HYG cassette (Split 1) was amplified using nested primer P7/P8. Similarly, fragment combining downstream *rad21*nc and the other half of the HYG cassette (Split 2) were prepared using primers P9/P10. For, *rec8* (FOXG_03390) same strategy was used for preparation in split 1 and split 2 cassettes. The primers used were enlisted in the Supplementary Table S2. For, random *HYG* (hygromycin B phosphotransferase) transformants full length HYG gene along with trpC promoter and terminator was amplified from pSilent1 using primers (P14 and P15).

*Fusarium oxysporum* protoplast preparation and PEG mediated transformation were done as described before (Di Pietro et al., 1998; Bae and Knudsen, 2000; Moradi et al., 2013; Ramamoorthy et al., 2015). Around 0.2–0.8 million protoplasts were mixed with ∼10 μg DNA of each cassette (split 1 and split 2). For fungal transformants selection top water agar layer was used with Hygromycin-B at final concentration 100 μg/mL. Transformation plates were kept for 4–5 days for transformants to appear. Each putative transformant was grown on PDB and conidia were spread again on selection plate to obtained monoconidial culture. Selected fungal transformants were screened using primers that amplify the full length of the target gene (primers P11/P12). Amplification of the *rad21*nc/*rec8* ORF fragment using P1/P11 and P13 primers was also done for further confirmation. In this case, true mutants do not show amplification of PCR product (Supplementary Figures S3, S4). Confirmed transformants were stored at -80°C and used for further experiments.

### Phenotypic Analysis for the *rad21*nc and *rec8* Mutants

To analyze the effect of different stress conditions on radial growth, equal size of mycelial agar plugs from each mutant culture were inoculated on PDA plates. The plates were incubated at 28°C for 3 days and then the diameter was measured. For sporulation analysis, mycelial agar plugs from WT and mutant cultures were inoculated in 5 mL PDB and incubated for 6 days at 28°C with shaking at 250 rpm. Spores were filtered and counted using a Neubauer counting chamber.

### Measurement of the Effect of Chromosome Stressors on Fungal Cultures

To measure the effect of hydroxyurea (HU) or benomyl on germination of WT and mutant strains, spores were isolated from the cultures grown on PDA plates at 28°C for 5–7 days by mere scrubbing of mycelia in 5 ml of water. Crude spore suspension was filtered, washed and dissolved in 1 ml of water. Spores were then inoculated for 14 h in PDB with or without treatment at 28°C, 250 rpm. The germinated spores were microscopically analyzed, using the five locations on a single side. Germinated and ungerminated spores were counted with the help of ImageJ software (Schneider et al., 2012). Experiments were repeated three times.

To measure the effect of DNA damage on colony formation of the different strains, conidia were pronged with serial dilutions onto PDA plates containing 0.01% of methyl methanesulfonate (MMS).

### Tomato Plant Infection Assays

Tomato seedling infection assays were conducted as described (Di Pietro et al., 1998, 2001) with some modifications. 15 days old tomato seedlings [Rehovot-13; (Katan and Ausher, 1974)] were dipped in 5 × 10^6^ spore per mL solution or in sterile water (mock) for 20 min. Then, plants were replanted in sterile soil-vermiculite mixture (60:40) and grown in a plant growth chamber at controlled growth conditions (25°C; ∼80% humidity; 14/10 h. light/dark cycle). Plants were monitored on a daily basis; survivors and dead plants were counted after 21 days post inoculation (dpi). Fisher’s exact test (two tailed) was used to assess the significance between the populations.

## Results

### Identifying Three *rad21* Paralog in Fusarium Species

**FIGURE 1:**
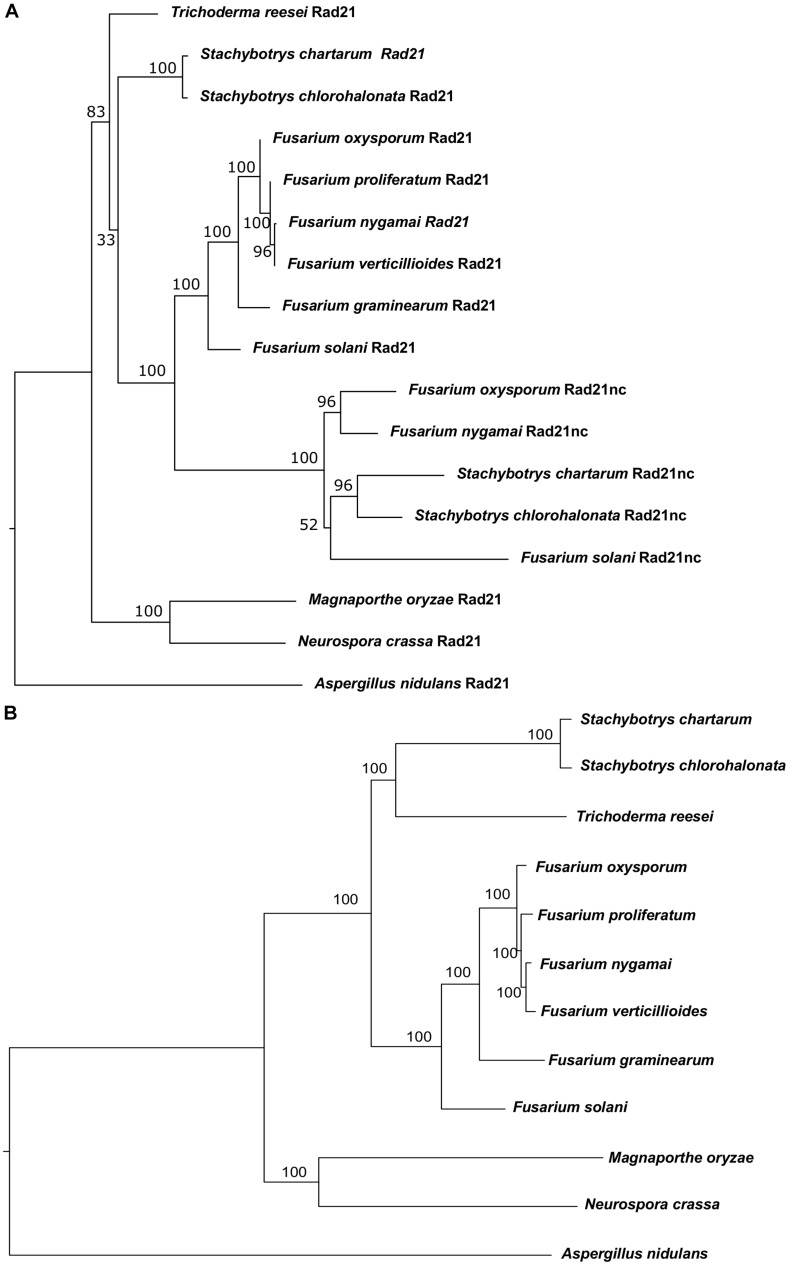
Phylogenetic analysis of Rad21 paralogs in Sordariomycetes. **(A)** Phylogenetic analysis of the protein sequences of *rad21* orthologs and paralogs from selected sordariomycetes fungi with *Aspergillus nidulans* serves as an outgroup of sordariomycetes. *N. crassa* and *Magnaporthe oryzae* serve as outgroup species for hypocreales. The tree was constructed as described under the Section “Materials and Methods.” **(B)** The phylogenetic tree of the species that were used to reconstruct the Rad21 tree was built using the genes described in Supplementary Table S1 as described in the Section “Materials and Methods.”

Initially, comparative genomics was used to identify *F. oxysporum* orthologs of proteins involved in DNA repair, recombination and chromosome transmission. *Saccharomyces cerevisiae* and *Aspergillus nidulans* proteins were collected from previously published sources (Goldman and Kafer, 2004; Stirling et al., 2011). We first identified orthologs of these genes in *Neurospora crassa* due to the quality of annotation and its short phylogenic divergence time from the Fusarium genus. Then, we used the *N. crassa* genes as seeds for BLASTP search in *F. oxysporum*. The BLASTP analysis of the *N. crassa* Rad21 protein against the proteome of *F. oxysporum* resulted in two paralogs with very high homology (*E*-value < 10^–153^) and another one that showed lower homology (*E*-value < 10^–10^). The two hits with the highest homology were the evolutionary conserved Rad21 and a never described before Rad21 paralog (FOXG_15850). The third paralog was Rec8. Rad21nc is not found in the closely related species to *F. oxysporum* and in other hypocreales but is found in *Fusarium solani* (XP_003044011), *Stachybotrys chartarum* (S7711_01263), *Stachybotrys chloronata*, and *Fusarium nygamai* (FNYG_15271) for this reason it was termed *rad21* non-conserved (*rad21*nc) (Figure 1A). To reveal the origin of Rad21nc we built a phylogenetic tree of Rad21 paralogs from several species in sordariomycetes. The Rad21 phylogenetic tree suggests that *rad21* gene duplication occurred after the divergence of *Trichoderma reesei* from the common ancestor of Fusarium, *Stachybotrys*, and *F. solani*. The duplication was followed by deletions of the gene that can not be found in *F. proliferatum*, *F. verticillioides*, *and F. graminearum*. An alternative, more complex, scenario is that the *rad21*nc gene was horizontally transferred from the branch of *Stachybotrys* to the branch of Fusarium species and then was lost from some of the Fusarium species. This scenario is supported by the fact that the *rad21*nc orthologs of *Stachybotrys* species are placed internally and not externally to branch of the Fusarium species (Figure 1A). Due to the low bootstrap values it is unclear if this is indeed the scenario (Figure 1A). In addition, the species tree that we built based on 90 genes (Supplementary Table S1) shows a slightly different phylogeny, especially with the placement of *T. reesei* and that of the *Stachybotrys* species. Therefore, it is hard to determine if indeed the gene duplication occurred after the divergence of *T. reesei*. This ambiguity is reflected in the low bootstrap values of the hypocreales tree as determined by other methods (Hongsanan et al., 2017). In any event, it is expected that *rad21*nc was found at the common ancestor of the Fusarium species. The most parsimonies evolutionary trajectory is that the *rad21*nc paralog was lost from most of the Fusarium species based on the fact that the Rad21nc sub-tree reflects the phylogenetic order of the species that contain the paralog (Figures 1A,B).

### Comparison of Rad21c and Rad21nc Protein Sequence With Yeast ScMcd1 (ScRad21)

**FIGURE 2:**
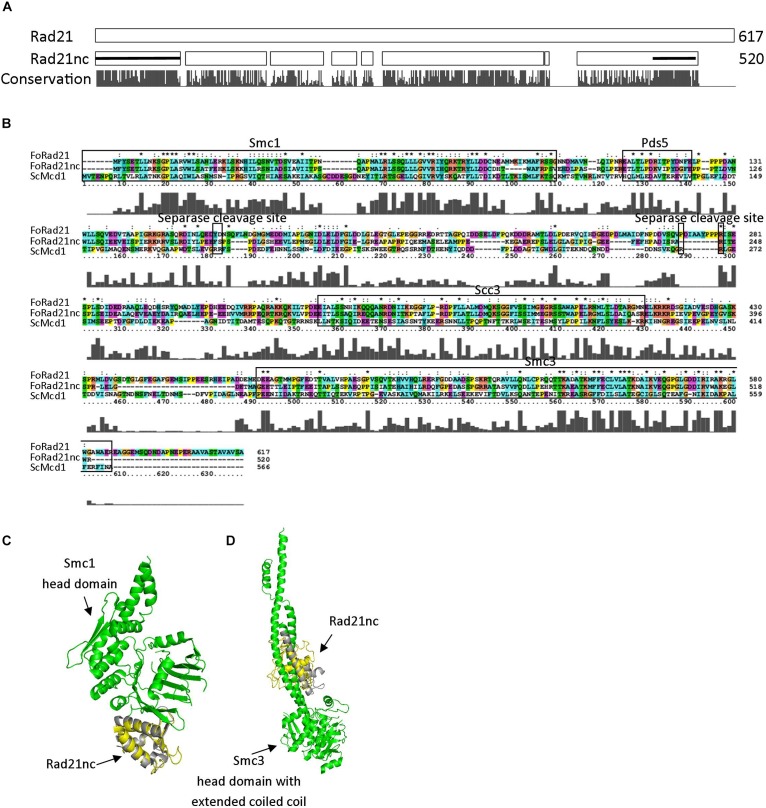
Rad21nc contains canonical eukaryal kleisin domains. **(A)** Sequence alignment of *F. oxysporum* canonical Rad21 (FOXG_00548) and Rad21nc (FOXG_15850) was done using ClustalX2. A schematic representation of the alignment is shown. The N-terminal domain (IPR006910) and the winged helix DNA-binding domain (IPR036390) in Rad21nc are marked by the black lines, respectively. The conservation level appears below as provided by the ClustalX2 algorithm. The full alignment is shown in Supplementary Figure S2. **(B)** Sequence alignment between *S. cerevisiae* ScMcd1 and *F. oxysporum* Rad21 and Rad21nc. The binding regions of *S. cerevisiae* ScMcd1 to Smc1, Pds5, Scc3, and Smc3 are indicated, as well as potential separase cleavage sites. **(C)** The structure of *F. oxysporum* Rad21nc N-terminal domain was predicted by I-TASSER (yellow). The structure was aligned on the crystal structure of the *S. cerevisiae* Smc1 head domain (green) bound to the N’ terminal fragment of ScMcd1 (gray) (1W1W). **(D)** The structure of *Fusarium oxysporum* Rad21nc C-terminal domain was predicted by I-TASSER (yellow). The structure was aligned on the crystal structure of the *S. cerevisiae* Smc3 head domain (green) bound to the C-terminal fragment of ScMcd1 (gray) (4UX3).

*rad21*nc is located on chromosome 8 of *F. oxysporum* f. sp. *lycopersici*; it is not part of the lineage specific loci of *F. oxysporum* f. sp. *lycopersici* and indeed the gene is encoded in all tested *F. oxysporum* spp. The gene, including UTR, is 2116 bp. The coding sequence is 1627 long bp and includes one 66 bp long intron. After examining all possible reading frames, we could not find out any known domain encoded within the intron. The sequences of Rad21 and Rad21nc were compared. Rad21nc is 97 amino acids shorter than the canonical Rad21 (Figure 2A and Supplementary Figure S2). The size difference between Rad21 and Rad21nc is due to several short internal gaps and a ∼30 amino acids C-terminal truncation. InterPro motif search revealed the characteristic Rad21/Rec8-like protein, N-terminal domain (IPR006910, a.a 1-82) and a winged helix DNA-binding domain (IPR036390, a.a 485-519) that mediate the interaction with Smc1 and Smc3, respectively, are marked by black line (Figure 2A). The interaction regions with Pds5 and Scc3 are also conserved while the sequence similarity outside these regions is lower (Figure 2B). Interestingly, scanning of Rad21nc against Eukaryotic Linear Motif resource (ELM) failed to identify the consensus separase cleavage motif. However, there are several partial motifs which may be recognized by the *F. oxysporum* separase protein. Based on the protein sequence, the structure of the N’ and C’ terminal domains of the Rad21nc protein were predicted by using the I-TASSER server. The predicted structure was aligned to the solved structures of *S. cerevisiae* Smc1 and Smc3 head domains that contain a N-terminal fragment of *S. cerevisiae* Mcd1 (Rad21). The structure of the N-terminal of Rad21nc is very similar to the ScMcd1 and completely overlaps with the ScMcd1 (Figure 2C). The Rad21nc C-terminal domain contains regions with uncertain folding. However, the helix that docks Rad21nc into Smc3 is well defined and properly localized into the coiled coil domain, when aligned to the solved structure of *S. cerevisiae* Smc3 with a C-terminal fragment of ScMcd1 (Figure 2D). This sequence and structural analyzes suggest that the fold of Rad21nc N’ and C’ terminal domain is similar to other kleisins and that the Rad21nc can most likely form a complex with Smc1 and Smc3 cohesin subunits.

### The RNA Epression of *rad21*c Gene Is Much Higher Than Both *rad21*nc and *rec8* Under All Tested Conditions

**FIGURE 3:**
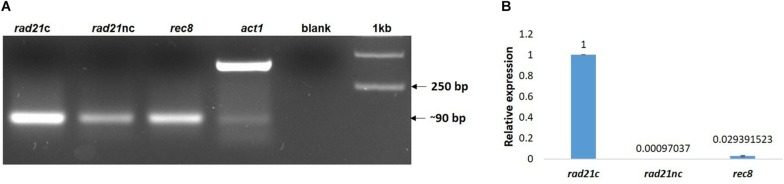
All *rad21* paralogs are expressed in germinating conidia but *rad21c* transcript level are much higher than *rad21c* or *rec8*. **(A)** RT-PCR analysis of the *rad21*c, *rad21*nc, *rec8*, and *act1* in germinating conidia of *F. oxysporum*. Paralog-specific *rad21* primers were used as described under the Section “Materials and Methods.” **(B)** Relative expression analysis of the *rad21*c, *rad21*nc, *rec8* transcripts in germinating conidia.

Next, we examined if the different *rad21* paralogs were expressed. RNA was purified from germinated spores of *F. oxysporum* f. sp. *lycopersici* and cDNA was prepared as described in the Section “Materials and Methods.” The cDNA was then amplified with paralog-specific primers. Figure 3A shows amplification of all three paralogs. Yet, quantitative assessment using qPCR of the different transcripts revealed that the expression of the *rec8* and *rad21*nc genes was 3% and even lower in comparison with *rad21*c (Figure 3B).

**FIGURE 4:**
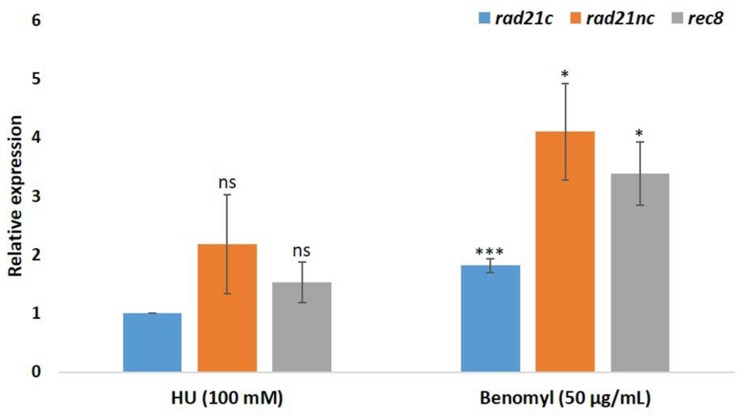
Benomyl exposure causes up-regulation of *rad21* paralogs. Average fold change are given for the *rad21*c, *rad21*nc, and *rec8* paralogs in 100 mM hydroxyurea (HU) or 50 μg/mL benomyl relative to PDB expression. Error bars represent the standard deviation between fold change expression of two independent experiments. ^*^ and ^∗∗∗^denote significant difference at *P* < 0.05 and *P* < 0.001, respectively, between PDB and test condition; ns, not significant.

We have also analyzed the RNA expression of *rad21* paralogs under HU or benomyl stress relative to PDB grown spores. qPCR analysis showed that *rad21*c, *rad21*nc, and *rec8* expression was slightly induced following benomyl stress Figure 4. The expression of *rad21*c was still much higher under treated condition relative to *rad21*nc or *rec8*.

### Hyphal Growth and Sporulation in the Δ*rad21*nc or Δ*rec8* Mutant Strains

Split marker approach was used to construct deletion cassettes for *rad21*nc or *rec8* genes as described in the Section “Materials and Methods.” Transformants were selected on hygromycin containing medium and PCR verified for *rad21*nc or *rec8* genes (Supplementary Figures S3, S4, respectively). Three confirmed independent mutants of Δ*rad21*nc (3, 10, and 18) and two independent Δ*rec8* mutants (1 and 12) were furthered used for the experiments below.

**FIGURE 5:**
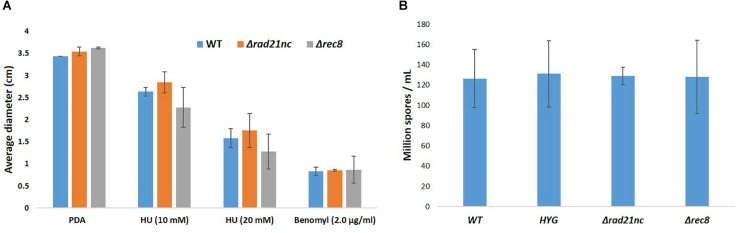
Δ*rad21*nc or Δ*rec8* mutant strains show WT level hyphal growth and sporulation. **(A)** Mycelial growth of WT and Δ*rad21*nc or Δ*rec8* mutants on PDA plates containing HU or Benomyl at different concentrations. Untreated hyphae were placed at center of PDA plates with or without the drugs; the average diameter of the colonies after 3 days of incubation is presented. **(B)** Spores from the different mutants and WT strains were isolated and counted after 6 days of growth on PDB as described in the Section “Materials and Methods.” Average spore count was calculated for three independent strains of each type used.

Mycelial growth and sporulation were analyzed for the mutants and control strains on PDA medium; no significant difference was found (Figures 5A,B). Next, mycelial growth of the mutants was also analyzed on PDA medium containing benomyl or HU. There was a slight decrease in the diameter of Δ*rec8* mutants in HU containing PDA plates as compared with WT strains but the growth difference was not significant (Figure 5A). No significant difference in the spore counts between WT, Δ*rad21*nc and Δ*rec8* strains was found (Figure 5B).

### Δ*rad21*nc or Δ*rec8* Mutants Exhibit Delayed Germination Under Cell Cycle Perturbations

**FIGURE 6:**
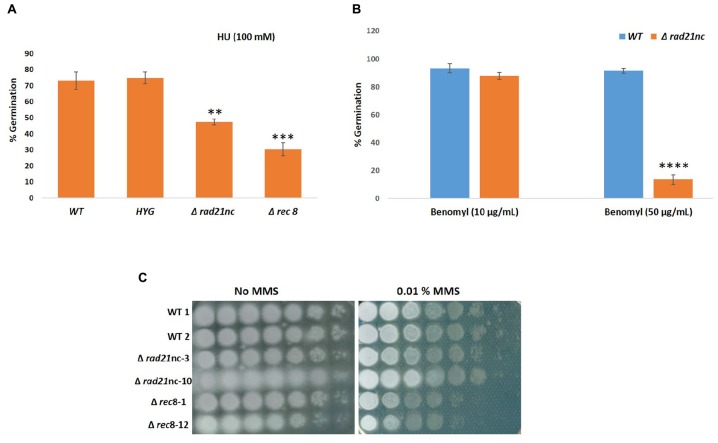
Conidia germination is inhibited in Δ*rad21*nc or Δ*rec8* strains during chromosome stress conditions. Percentage of germination of fungal spores in PDB containing HU 100 mM **(A)**, Benomyl 10 μg/ml and 50 μg/ml **(B)**. Average of three experiments using two biological replicates of each strain in each experiment is shown. The average of counts of five frames for each biological repeat is presented. **(C)** Spores of WT, Δ*rad21*nc, and Δ*rec8* strains were serially diluted and spotted on PDA plates with or without 0.01% MMS. ^∗∗^, ^∗∗∗^, and ^∗∗∗∗^ denote significant difference at *P* < 0.01, *P* < 0.001, and *P* < 0.0001 level, respectively, between wild-type and mutants; ns, not significant.

We analyzed the Δ*rad21*nc or Δ*rec8* mutant spore germination in different conditions. The germination of spores was measured in PDB medium as described under the Section “Materials and Methods”; no significant difference was observed between WT and the mutants. Spore germination under exposure of the mitosis inhibitor benomyl and the DNA replication inhibitor HU was measured. The Δ*rad21*nc and Δ*rec8* strains showed significantly lower germination rate under the 100 mM HU in PDB. The average percentage germination of WT fungal strains was 73.03% and *HYG* transformed strains it was 74.82%. Germination rate under these conditions was only 47.36% in Δ*rad21*nc and 30.36% in Δ*rec8* strains (Figure 6A). Similarly, when conidia were treated with 50 μg/ml benomyl, average percentage germination in WT strains was 91.46% and it was reduced to 13.68% in the Δ*rad21*nc mutant Δ*rec8* mutant showed inconsistent results (Figure 6B). In conclusion, we observed for Δ*rad21*nc and to lesser extent Δ*rec8* phenotypes that are directly linked to cell cycle perturbation. These results are in agreement with the notion that Rec8 and Rad21nc function in alternative cohesin complexes since cohesin mutants are sensitive to benomyl and HU (Guacci et al., 1997; Aguilar et al., 2005; Heidinger-Pauli et al., 2010). We measured the expression of the different *rad21* paralogs under the same conditions of chromosome stress in the Δ*rad21*nc or Δ*rec8* strains. No dramatic increase in the expression of any of the paralogs was detected in the mutants comparing with WT strains. Small differences cannot be overruled due to the very low transcript levels of *rad21*nc and *rec8* (data not shown).

Sensitivity toward the DNA damaging agent MMS was analyzed for WT, Δ*rad21*nc, or Δ*rec8* strains in a spot assay using serial dilutions (Figure 6C). Δ*rec8* mutant strains were more sensitive than WT to MMS by at least an order of magnitude. No sensitivity was observed in the Δ*rad21*nc mutant. Mutations in cohesin subunits are expected to increase mutagenicity. Measuring the rate of resistance to benomyl is a common forward mutation assay in fungi. We studied the rate of benomyl resistant mutant formation in WT, Δ*rad21*nc, and Δ*rec8* strains. Nine cultures of each strain were grown in PDB for 5 days. Next, spores were collected and spread with appropriate dilutions on PDA plates and PDA plates containing benomyl (2 μg/ml). Colonies were counted 2–3 days (PDA) or 6–7 days (benomyl) after plating. The rate of resistant mutants was calculated as previously described (Covo et al., 2014). While the median rates of WT and Δ*rec8* strains were similar, the rate of the Δ*rad21*nc was about 20 fold higher (*P*-value = 0.02 two tails *T*-test, Table 1).

**TABLE 1:** Increased rate of benomyl resistance inΔ*rad21*nc strains.

**Genotype**	**Median Benomyl resistance rate**	***P*-value** **(*T*-test WT: *rad21* mutant)**
WT	2 × 10^–6^	ND
Δ*rad21*nc	44 × 10^–6^	0.03
Δ*rec8*	4 × 10^–6^	0.48

### Pathological Analysis of the Δ*rad21*nc or Δ*rec8* Mutant Strains

**FIGURE 7:**
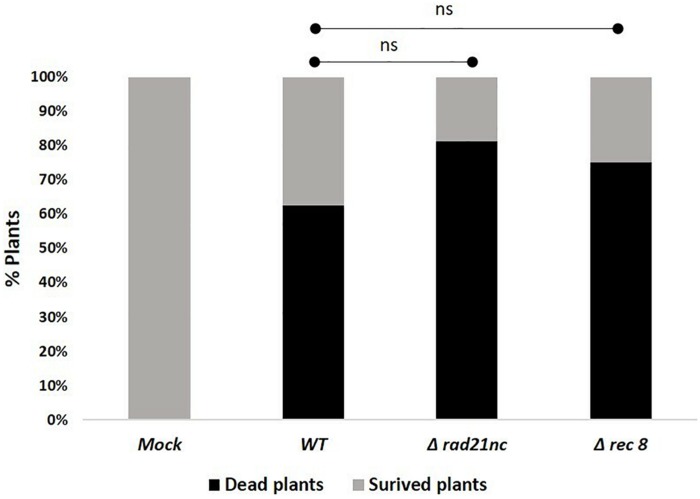
*rad21*nc or *rec8* genes are not required for generation of wilt disease in tomatoes. Tomato seedlings infection using the Mock- water control, WT- WT, Δ*rad21*nc, or Δ*rec8* strain. *Fusarium* wilt disease severity was determined by measuring the percentage of dead and survived plants after 21 days post inoculation (dpi); ns, not significant.

Finally, we determined the effect of a mutation in *rad21nc* or *rec8* on the ability of *F. oxysporum* f. sp. *lycopersici* to cause wilt disease in tomatoes. Plant infection was done using Δ*rad21*nc or Δ*rec8* strains on tomato seedlings. After 21 days post inoculation (dpi), we did not observe any significant change in the percentage of dead plants between the WT and mutant strains Figure 7 and Supplementary Figure S5.

## Discussion

Rad21 as part of cohesin is essential for cell division and faithful transmission of chromosomes. It is also important for mitotic homologous recombination and gene expression (Onn et al., 2008). The model ascomycete fungi *S. cerevisiae*, *Schizosaccharomyces pombe*, *N. crassa*, and *A. nidulans* and most other sequenced species have two paralogs of *rad21*, one of them, *rec8* is supposed to function in homologous recombination during meiosis (Nasmyth and Haering, 2005, 2009). In some organisms there are more than two *rad21* paralogs; in all examined cases these paralogs had at least some non-overlapping functions (Bhatt et al., 1999; Severson et al., 2009; Ishiguro et al., 2011; da Costa-Nunes et al., 2014; Severson and Meyer, 2014). In an attempt to identify *F. oxysporum*-specific chromosome biology proteins we have found a non-conserved Rad21 paralog in few hypocreales species. Due to the small number of species that encode for Rad21nc it is hard to describe its evolution trajectory in high confidence. However, evidence lead to scenario of gene duplication after the divergence of *Trichoderma* species and before the divergence of *Stachybotrys* species from *F. solani* – followed by loss of the gene in most Fusarium species. This is based on the fact that the Rad21nc is found both in *F. oxysporum* and *F. nygamai* which is part of the *F. fujikuroi* species complex (Figure 1). The comparison of the sequences between the conserved and non-conserved *rad21* paralogs shows that the non-conserved one retains some classic α-kleisins domains (Smc1, Smc3 binding) (Figure 2). This may indicate that Rad21nc functions as part of a cohesion complex.

We analyzed the deletion strains of *rad21*nc or *rec8* gene. Δ*rad21*nc or Δ*rec8* mutant were similar to WT regarding sporulation and radial growth. We observed decrease in germination of the spores under the cell cycle stresses (Figure 6). HU induces DNA replication stress while benomyl activates the G2/M checkpoint. The sensitivity of *rad21*nc to these drugs suggests that *rad21*nc supports chromosome transmission or functions in a chromosome transmission checkpoint response. The germination of Δ*rec8* strains under HU stress and colony formation during MMS exposure are lower than WT cells suggesting a role in homologous recombinational repair as recently described for Rec8 from *Ustilago maydis* (de Sena-Tomás et al., 2011; Sutherland and Holloman, 2018). Yet, a role in cell cycle control and non homologous recombination is also possible. The phenotypes of Δ*rec8* and Δ *rad21*nc strains are surprising since the expression of the two genes is very low even under HU and benomyl exposures (Figure 4). A probable explanation to how low amounts of a cohesin subunit may affect cells could be the fact that Rad21nc does not encode for a full separase domain. It is possible that few cohesin molecules associated with Rad21nc are evicted from chromosome in an alternative way that is needed for proper chromosome segregation in *F. oxysporum* under unique conditions.

Further studies are very much required to analyze the specific roles of the different Rad21 paralogs in fungal life cycle. Understanding the role of alternative cohesin complexes in *F. oxysporum* and other species will further open new dimensions for understanding the pathogen population genetics and genome evolution.

## Author Contributions

SC, MP, and YA designed the project and collected the initial data. SC, MP, and VB conducted the experiments. SC, MP, EH-C, and IO analyzed the data. SC, MP, and IO wrote the manuscript.

## Conflict of Interest Statement

The authors declare that the research was conducted in the absence of any commercial or financial relationships that could be construed as a potential conflict of interest.

## Supplementary Material

FIGURE S1 A diagram explaining the strategy for the generation of the split marker cassette of *rad21*nc or *rec8*. The primer IDs’ match the ones provided in Supplementary Table S2.

FIGURE S2 Sequence alignment of *F. oxysporum* Rad21 and Rad21nc. Alignment was generated by using ClustalX2.

FIGURE S3 Screening for Δ*rad21*nc transformants. **(A)** Confirmation of disruption of the *rad21*nc open reading frame using hygromycin resistant cassettes. PCR was used to amplify full length locus in wild type (WT) (2.0 kb), and the deleted locus (2.3 kb) using the P11 and P12 primers. 1 kb–1 kb marker (GeneDirex), WT- untransformed control, 1-18 are putative fungal transformants. **(B)** Amplification of the *rad21*nc ORF fragment using P1 and P13 primers, true mutants do not show amplification. 1 kb–1 kb marker (GeneDirex), W1, W2, and W3- untransformed control, 1-18 are putative fungal transformants. Transformants 3, 5, 6, 10, and 18 are true deleted strains for *rad21*nc locus.

FIGURE S4 Screening for Δ*rec8* transformants. Confirmation of disruption of the *rec8* open reading frame using hygromycin resistant cassettes **(A)** Amplification of full length *rec8* locus in WT (2.5 kb) and the deleted locus (2.1 kb) using the P11 and P12 primers. 1 kb–1 kb marker (GeneDirex), W1 and W2 – untransformed control, 1-20 are putative fungal transformants. **(B)** Amplification of the *rec8* ORF using P11 and P13 primers, true mutants do not show amplification. 1 kb–1 kb marker (GeneDirex), W1 and W2 – untransformed control, 1-20 are putative fungal transformants. Transformants 1 and 12 are true deleted strains for *rec8* locus.

FIGURE S5 Tomato wilt disease caused by *F. oxysporum* strains mutated in *rec8* or *rad21*nc. Tomato plants infected with WT, Δ*rad21*nc, or Δ*rec8* fungal strains and water control (mock) after 21 dpi under controlled growth condition as described under the Section “Materials and Methods.”

TABLE S1 90 DNA Repair proteins that were used to build the species tree that is presented in Figure 1.

TABLE S2 Primers used in the study.

## References

[B1] Aguilar C. Davidson C. Dix M. Stead K. Zheng K. Hartman T. (2005). Topoisomerase II suppresses the temperature sensitivity of Saccharomyces cerevisiae pds5 mutants, but not the defect in sister chromatid cohesion. *Cell Cycle* 4 1294–1304. 10.4161/cc.4.9.1997 16096371

[B2] Bae Y. S. Knudsen G. R. (2000). Cotransformation of *Trichoderma harzianum* with beta -Glucuronidase and Green Fluorescent Protein genes provides a useful tool for monitoring fungal growth and activity in natural soils. *Appl. Environ. Microbiol.* 66 810–815. 10.1128/aem.66.2.810-815.2000 10653755 PMC91900

[B3] Bai X. Peirson B. N. Dong F. Xue C. Makaroff C. A. (1999). Isolation and characterization of SYN1, a RAD21 -like gene essential for meiosis in *Arabidopsis*. *Plant Cell* 11 417–430. 10.1105/tpc.11.3.417 10072401 PMC144192

[B4] Bhatt A. M. Lister C. Page T. Fransz P. Findlay K. Jones G. H. (1999). The DIF1 gene of *Arabidopsis* is required for meiotic chromosome segregation and belongs to the REC8/RAD21 cohesin gene family. *Plant J.* 19 463–472. 10.1046/j.1365-313x.1999.00548.x 10504568

[B5] Bolaños-Villegas P. De K. Pradillo M. Liu D. Makaroff C. A. (2017). In favor of establishment: regulation of chromatid cohesion in plants. *Front. plant sci.* 8:846. 10.3389/fpls.2017.00846 28588601 PMC5440745

[B6] Bose T. Gerton J. L. (2010). Cohesinopathies, gene expression, and chromatin organization. *J. Cell Biol.* 189 201–210. 10.1083/jcb.200912129 20404106 PMC2856913

[B7] Catlett N. L. Lee B.-N. Yoder O. C. Turgeon B. G. (2003). Split-Marker recombination for efficient targeted deletion of fungal genes. *Fungal Genet. Newsl.* 50 9–11. 10.4148/1941-4765.1150

[B8] Covo S. Ma W. Westmoreland J. W. Gordenin D. A. Resnick M. A. (2012). Understanding the origins of UV-induced recombination through manipulation of sister chromatid cohesion. *Cell Cycle* 11 3937–3944. 10.4161/cc.21945 22987150 PMC3507489

[B9] Covo S. Puccia C. M. Argueso J. L. Gordenin D. A. Resnick M. A. (2014). The sister chromatid cohesion pathway suppresses multiple chromosome gain and chromosome amplification. *Genetics* 196 373–384. 10.1534/genetics.113.159202 24298060 PMC3914611

[B10] Covo S. Westmoreland J. W. Gordenin D. A. Resnick M. A. (2010). Cohesin is limiting for the suppression of dna damage–induced recombination between homologous chromosomes. *PLoS Genet.* 6:e1001006. 10.1371/journal.pgen.1001006 20617204 PMC2895640

[B11] da Costa-Nunes J. A. Bhatt A. M. O’Shea S. West C. E. Bray C. M. Grossniklaus U. (2006). Characterization of the three *Arabidopsis thaliana* RAD21 cohesins reveals differential responses to ionizing radiation. *J. Exp. Bot.* 57 971–983. 10.1093/jxb/erj083 16488915

[B12] da Costa-Nunes J. A. Capitão C. Kozak J. Costa-Nunes P. Ducasa G. M. Pontes O. (2014). The AtRAD21.1 *and AtRAD*21.3 *Arabidopsis* cohesins play a synergistic role in somatic DNA double strand break damage repair. *BMC Plant Biol.* 14:353. 10.1186/s12870-014-0353-9 25511710 PMC4273318

[B13] de Sena-Tomás C. Fernández-Álvarez A. Holloman W. K. Pérez-Martín J. (2011). The DNA damage response signaling cascade regulates proliferation of the phytopathogenic fungus Ustilago maydis in planta. *Plant Cell* 23 1654–1665. 10.1105/tpc.110.082552 21478441 PMC3101559

[B14] Dean R. Dean R. Van Kan J. A. Pretorius Z. A. Hammond-Kosack K. E. Di Pietro A. (2012). The Top 10 fungal pathogens in molecular plant pathology. *Mol. Plant Pathol* 13 414–430. 10.1111/j.1364-3703.2011.00783.x 22471698 PMC6638784

[B15] Di Pietro A. García-MacEira F. I. Méglecz E. Roncero M. I. (2001). A MAP kinase of the vascular wilt fungus *Fusarium oxysporum* is essential for root penetration and pathogenesis. *Mol. Microbiol.* 39 1140–1152. 10.1111/j.1365-2958.2001.02307.x 11251832

[B16] Di Pietro A. Isabel M. Roncero G. (1998). Cloning, expression, and role in pathogenicity of pg1 encoding the major extracellular endopolygalacturonase of the vascular wilt pathogen *Fusarium oxysporum*. *Mol. Plant Microbe Interact* 11 91–98. 10.1094/mpmi.1998.11.2.91 9450333

[B17] Dong F. Cai X. Makaroff C. A. (2001). Cloning and characterization of two *Arabidopsis* genes that belong to the RAD21/REC8 family of chromosome cohesin proteins. *Gene* 271 99–108. 10.1016/s0378-1119(01)00499-1 11410371

[B18] Gligoris T. G. Scheinost J. C. Bürmann F. Petela N. Chan K. L. Uluocak P. (2014). Closing the cohesin ring: structure and function of its Smc3-kleisin interface. *Science* 346 963–967. 10.1126/science.1256917 25414305 PMC4300515

[B19] Goldman G. H. Kafer E. (2004). Aspergillus nidulans as a model system to characterize the DNA damage response in eukaryotes. *Fungal Genet. Biol.* 41 428–442. 10.1016/j.fgb.2003.12.001 14998526

[B20] Guacci V. Koshland D. Strunnikov A. (1997). A direct link between sister chromatid cohesion and chromosome condensation revealed through the analysis of MCD1 in S. *cerevisiae*. *Cell* 91 47–57. 10.1016/s0092-8674(01)80008-8 9335334 PMC2670185

[B21] Haering C. H. Löwe J. Hochwagen A. Nasmyth K. (2002). Molecular architecture of SMC proteins and the yeast cohesin complex. *Mol. Cell* 9 773–788. 10.1016/s1097-2765(02)00515-4 11983169

[B22] Haering C. H. Schoffnegger D. Nishino T. Helmhart W. Nasmyth K. Löwe J. (2004). Structure and Stability of Cohesin’s Smc1-Kleisin Interaction. *Mol. Cell* 15 951–964. 10.1016/j.molcel.2004.08.030 15383284

[B23] Heidinger-Pauli J. M. Mert O. Davenport C. Guacci V. Koshland D. (2010). Systematic reduction of cohesin differentially affects chromosome segregation, condensation, and DNA repair. *Curr. Biol.* 20 957–963. 10.1016/j.cub.2010.04.018 20451387 PMC2892909

[B24] Hongsanan S. Maharachchikumbura S. S. Hyde K. D. Samarakoon M. C. Jeewon R. Zhao Q. (2017). An updated phylogeny of Sordariomycetes based on phylogenetic and molecular clock evidence. *Fungal Divers.* 84 25–41. 10.1007/s13225-017-0384-2

[B25] Ishiguro K. Kim J. Fujiyama-Nakamura S. Kato S. Watanabe Y. (2011). A new meiosis-specific cohesin complex implicated in the cohesin code for homologous pairing. *EMBO Rep.* 12 267–275. 10.1038/embor.2011.2 21274006 PMC3059921

[B26] Kagey M. H. Newman J. J. Bilodeau S. Zhan Y. Orlando D. A. van Berkum N. L. (2010). Mediator and cohesin connect gene expression and chromatin architecture. *Nature* 467 430–435. 10.1038/nature09380 20720539 PMC2953795

[B27] Kakui Y. Uhlmann F. (2018). SMC complexes orchestrate the mitotic chromatin interaction landscape. *Curr. Genet.* 64 335–339. 10.1007/s00294-017-0755-y 28936767 PMC5851691

[B28] Katan J. Ausher R. (1974). Distribution of race 2 of *Fusarium oxysporum* f. *sp.lycopersici in tomato fields in Israel*. *Phytoparasitica* 2 83–90. 10.1007/bf02980292

[B29] Katoh K. Asimenos G. Toh H. (2009). “Multiple Alignment of DNA Sequences with MAFFT,” in *Bioinformatics for DNA Sequence Analysis. Methods in Molecular Biology (Methods and Protocols)*, Vol. 537 ed. Posada D. (New York, NY: Humana Press), 39–64. 10.1007/978-1-59745-251-9_3 19378139

[B30] Kim K. P. Weiner B. M. Zhang L. Jordan A. Dekker J. Kleckner N. (2010). Sister cohesion and structural axis components mediate homolog bias of meiotic recombination. *Cell* 143 924–937. 10.1016/j.cell.2010.11.015 21145459 PMC3033573

[B31] Larkin M. A. Blackshields G. Brown N. P. Chenna R. Chenna R. McGettigan P. A. (2007). Clustal W and Clustal X version 2.0. *Bioinformatics* 23 2947–2948. 10.1093/bioinformatics/btm404 17846036

[B32] Lengronne A. Katou Y. Mori S. Yokobayashi S. Kelly G. P. Itoh T. (2004). Cohesin relocation from sites of chromosomal loading to places of convergent transcription. *Nature* 430 573–578. 10.1038/nature02742 15229615 PMC2610358

[B33] Li Y. Huang W. Niu L. Umbach D. M. Covo S. Li L. (2013). Characterization of constitutive CTCF/cohesin loci: a possible role in establishing topological domains in mammalian genomes. *BMC Genomics* 14:553. 10.1186/1471-2164-14-553 23945083 PMC3765723

[B34] Livak K. J. Schmittgen T. D. (2001). Analysis of relative gene expression data using real-time quantitative PCR and the 2- ΔΔCT method. *Methods* 25 402–408. 10.1006/meth.2001.1262 11846609

[B35] Ma L. J. Does H. C. van der Borkovich K. A. Coleman J. J. Marie-Josée D. Pietro V. (2010). Comparative genomics reveals mobile pathogenicity chromosomes in *Fusarium*. *Nature* 464 367–373. 10.1038/nature08850 20237561 PMC3048781

[B36] Ma L. J. Geiser D. M. Proctor R. H. Rooney A. P. O’Donnell K. Trail F. (2013). Fusarium pathogenomics. *Annu. Rev. Microbiol.* 67 399–416. 10.1146/annurev-micro-092412-155650 24024636

[B37] Moradi S. Sanjarian F. Safaie N. Mousavi A. Reza G. Khaniki B. (2013). A modified method for transformation of *Fusarium graminearum*. *J. Crop Prot.* 2 297–304. 10.1016/j.fgb.2012.05.008 22664277

[B38] Nakayashiki H. Hanada S. Quoc N. B. Kadotani N. Tosa Y. Mayama S. (2005). RNA silencing as a tool for exploring gene function in ascomycete fungi. *Fungal Genet. Biol.* 42 275–283. 10.1016/j.fgb.2005.01.002 15749047

[B39] Nasmyth K. Haering C. H. (2005). The structure and function of Smc and Kleisin complexes. *Annu. Rev. Biochem.* 74 595–648. 10.1146/annurev.biochem.74.082803.133219 15952899

[B40] Nasmyth K. Haering C. H. (2009). Cohesin: its roles and mechanisms. *Annu. Rev. Genet.* 43 525–558. 10.1146/annurev-genet-102108-134233 19886810

[B41] Onn I. Heidinger-Pauli J. M. Guacci V. Ünal E. Koshland D. E. (2008). Sister chromatid cohesion: a simple concept with a complex reality. *Annu. Rev. Cell Dev. Biol.* 24 105–129. 10.1146/annurev.cellbio.24.110707.175350 18616427

[B42] Palecek J. J. Gruber S. (2015). Kite Proteins: a superfamily of smc/kleisin partners conserved across bacteria, archaea, and eukaryotes. *Structure* 23 2183–2190. 10.1016/j.str.2015.10.004 26585514

[B43] Ramamoorthy V. Govindaraj L. Dhanasekaran M. Vetrivel S. Kumar K. K. Ebenezar E. (2015). Combination of driselase and lysing enzyme in one molar potassium chloride is effective for the production of protoplasts from germinated conidia of Fusarium verticillioides. *J. Microbiol. Methods* 111 127–134. 10.1016/j.mimet.2015.02.010 25724844

[B44] Roy A. Kucukural A. Zhang Y. (2010). I-TASSER: a unified platform for automated protein structure and function prediction. *Nat. Protoc.* 5 725–738. 10.1038/nprot.2010.5 20360767 PMC2849174

[B45] Schneider C. A. Rasband W. S. Eliceiri K. W. (2012). NIH Image to ImageJ: 25 years of image analysis. *Nat. Methods* 9 671–675. 10.1038/nmeth.2089 22930834 PMC5554542

[B46] Sela I. Ashkenazy H. Katoh K. Pupko T. (2015). GUIDANCE2: accurate detection of unreliable alignment regions accounting for the uncertainty of multiple parameters. *Nucleic Acids Res.* 43 W7–W14. 10.1093/nar/gkv318 25883146 PMC4489236

[B47] Severson A. F. Ling L. van Zuylen V. Meyer B. J. (2009). The axial element protein HTP-3 promotes cohesin loading and meiotic axis assembly in C. *elegans to implement the meiotic program of chromosome segregation*. *Genes Dev.* 23 1763–1778. 10.1101/gad.1808809 19574299 PMC2720254

[B48] Severson A. F. Meyer B. J. (2014). Divergent kleisin subunits of cohesin specify mechanisms to tether and release meiotic chromosomes. *eLife* 3:e03467. 10.7554/eLife.03467 25171895 PMC4174578

[B49] Sjögren C. Nasmyth K. (2001). Sister chromatid cohesion is required for postreplicative double-strand break repair in *Saccharomyces cerevisiae*. *Curr. Biol.* 11 991–995. 10.1016/s0960-9822(01)00271-8 11448778

[B50] Spencer F. Gerring S. L. Connelly C. Hieter P. (1990). Mitotic chromosome transmission fidelity mutants in *Saccharomyces cerevisiae*. *Genetics* 124 237–249. 2407610 10.1093/genetics/124.2.237 PMC1203917

[B51] Stamatakis A. (2014). RAxML version 8: a tool for phylogenetic analysis and post-analysis of large phylogenies. *Bioinformatics* 30 1312–1313. 10.1093/bioinformatics/btu033 24451623 PMC3998144

[B52] Stirling P. C. Bloom M. S. Solanki-Patil T. Smith S. Sipahimalani P. Li Z. (2011). The complete spectrum of yeast chromosome instability genes identifies candidate CIN cancer genes and functional roles for ASTRA complex components. *PLoS Genet.* 7:e1002057. 10.1371/journal.pgen.1002057 21552543 PMC3084213

[B53] Sutherland J. H. Holloman W. K. (2018). Loss of cohesin subunit Rec8 switches Rad51 mediator dependence in resistance to formaldehyde toxicity in *Ustilago maydis*. *Genetics* 210 559–572. 10.1534/genetics.118.301439 30082279 PMC6216591

[B54] Uhlmann F. Lottspeich F. Nasmyth K. (1999). Sister-chromatid separation at anaphase onset is promoted by cleavage of the cohesin subunit Scc1. *Nature* 400 37–42. 10.1038/21831 10403247

[B55] Ünal E. Arbel-Eden A. Sattler U. Shroff R. Lichten M. Haber J. E. (2004). dna damage response pathway uses histone modification to assemble a double-strand break-specific cohesin domain. *Mol. Cell* 16 991–1002. 10.1016/j.molcel.2004.11.027 15610741

[B56] van Ruiten M. S. Rowland B. D. (2018). SMC complexes: universal DNA looping machines with distinct regulators. *Trends Genet.* 34 477–487. 10.1016/j.tig.2018.03.003 29606284

[B57] Vlaardingerbroek I. Beerens B. Rose L. Fokkens L. Cornelissen B. J. C. Rep M. (2016). Exchange of core chromosomes and horizontal transfer of lineage-specific chromosomes in *Fusarium oxysporum*. *Environ. Microbiol.* 18 3702–3713. 10.1111/1462-2920.13281 26941045

[B58] Watanabe Y. Nurse P. (1999). Cohesin Rec8 is required for reductional chromosome segregation at meiosis. *Nature* 400 461–464. 10.1038/22774 10440376

[B59] Yang J. Yan R. Roy A. Xu D. Poisson J. Zhang Y. (2015). The I-TASSER suite: protein structure and function prediction. *Nat. Methods* 12 7–8. 10.1038/nmeth.3213 25549265 PMC4428668

[B60] Yang Z. (1994). Maximum likelihood phylogenetic estimation from DNA sequences with variable rates over sites: approximate methods. *J. Mol. Evol.* 39 306–314. 10.1007/BF00160154 7932792

[B61] Yu J.-H. Hamari Z. Han K.-H. Seo J.-A. Reyes-Domínguez Y. Scazzocchio C. (2004). Double-joint PCR: a PCR-based molecular tool for gene manipulations in filamentous fungi. *Fungal Genet. Biol.* 41 973–981. 10.1016/j.fgb.2004.08.001 15465386

[B62] Zhang Y. (2008). I-TASSER server for protein 3D structure prediction. *BMC Bioinformatics* 9:40. 10.1186/1471-2105-9-40 18215316 PMC2245901

[B63] Zickler D. Kleckner N. (1999). Meiotic chromosomes: integrating structure and function. *Ann. Rev. Genet.* 33 603–754. 10.1146/annurev.genet.33.1.603 10690419

