# Supplementary figures and images for: Alternative Functional rad21 Paralogs in Fusarium oxysporum
Source: Front Microbiol. 2019 Jun 18;10:1370. doi: 10.3389/fmicb.2019.01370 (PMC6591460; doi:10.3389/fmicb.2019.01370)

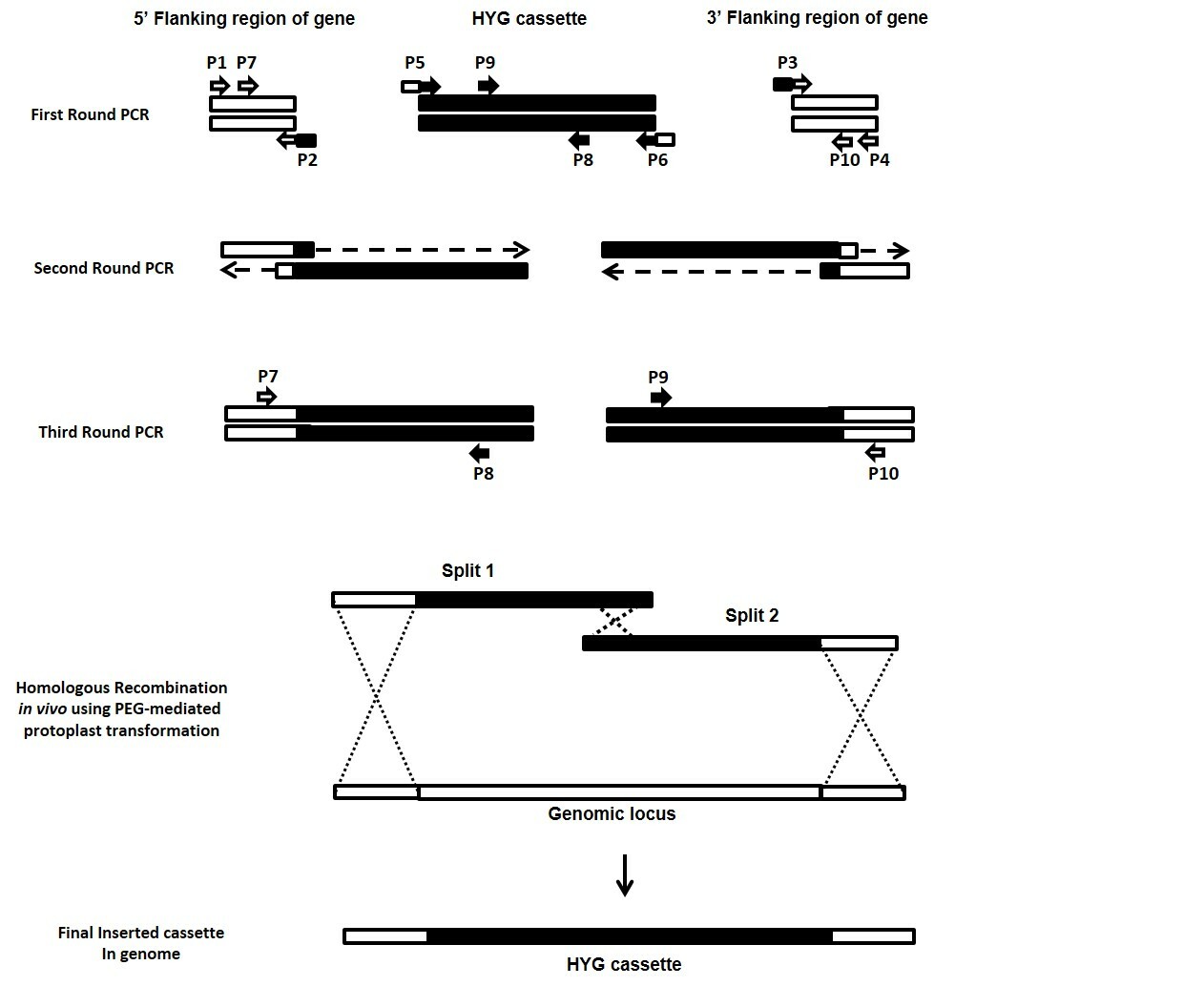

Supplement: FIGURE S1 — A diagram explaining the strategy for the generation of the split marker cassette of rad21nc or rec8. The primer IDs’ match the ones provided in Supplementary Table S2. [file Image_1.JPEG]

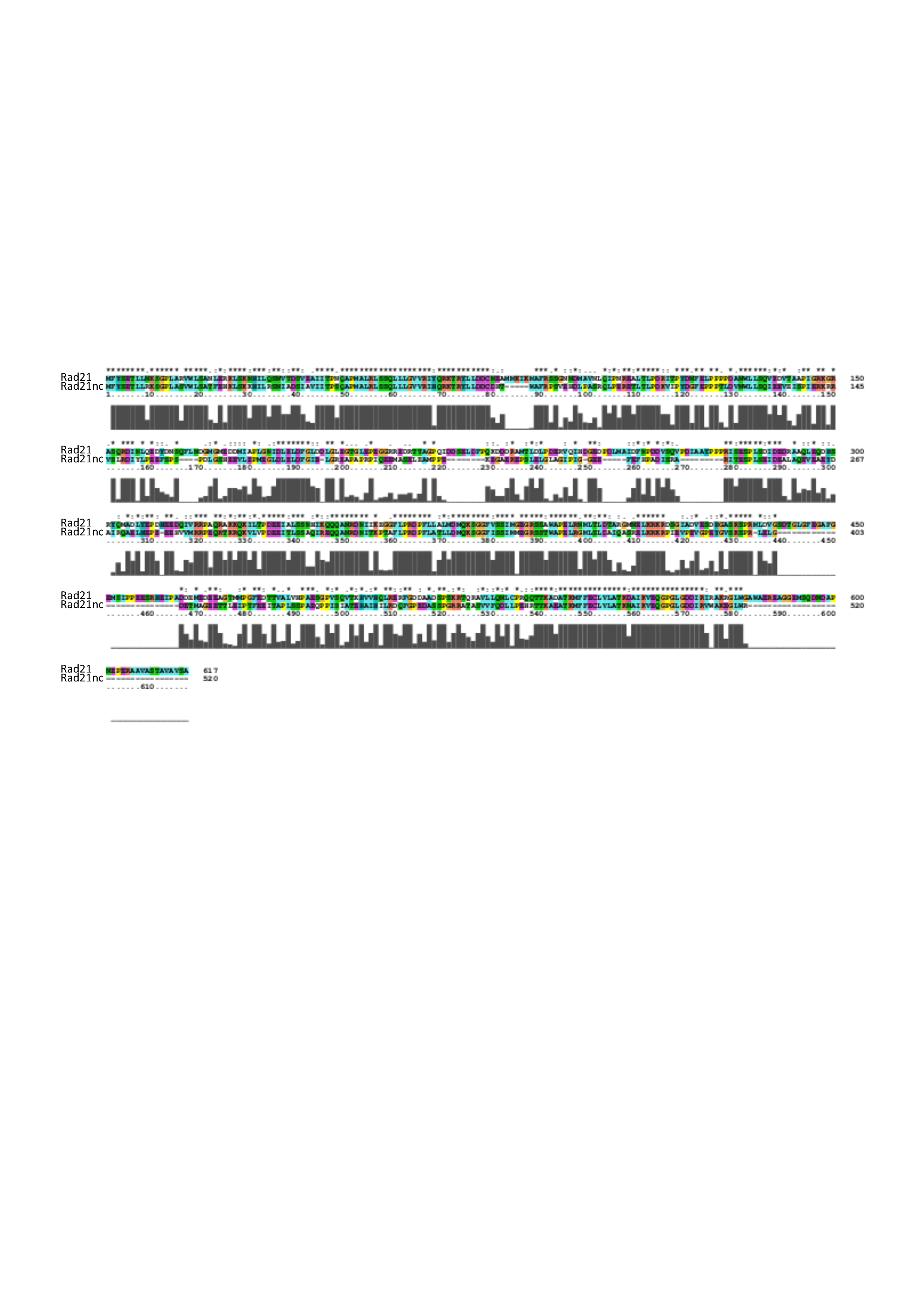

Supplement: FIGURE S2 — Sequence alignment of F. oxysporum Rad21 and Rad21nc. Alignment was generated by using ClustalX2. [file Image_2.TIF]

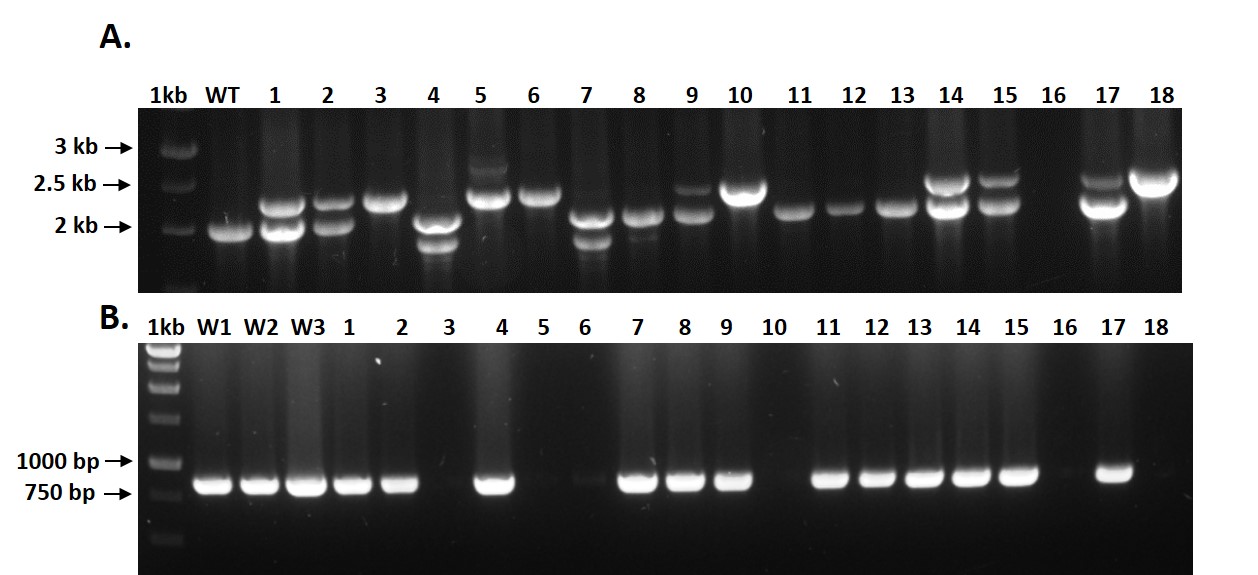

Supplement: FIGURE S3 — Screening for Δrad21nc transformants. (A) Confirmation of disruption of the rad21nc open reading frame using hygromycin resistant cassettes. PCR was used to amplify full length locus in wild type (WT) (2.0 kb), and the deleted locus (2.3 kb) using the P11 and P12 primers. 1 kb–1 kb marker (GeneDirex), WT- untransformed control, 1-18 are putative fungal transformants. (B) Amplification of the rad21nc ORF fragment using P1 and P13 primers, true mutants do not show amplification. 1 kb–1 kb marker (GeneDirex), W1, W2, and W3- untransformed control, 1-18 are putative fungal transformants. Transformants 3, 5, 6, 10, and 18 are true deleted strains for rad21nc locus. [file Image_3.JPEG]

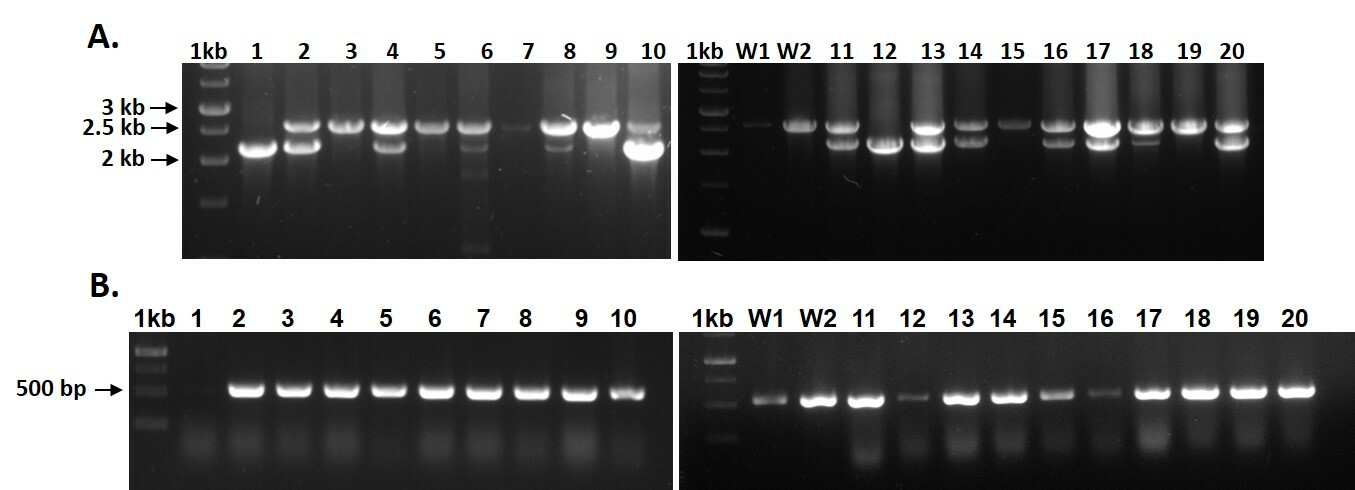

Supplement: FIGURE S4 — Screening for Δrec8 transformants. Confirmation of disruption of the rec8 open reading frame using hygromycin resistant cassettes (A) Amplification of full length rec8 locus in WT (2.5 kb) and the deleted locus (2.1 kb) using the P11 and P12 primers. 1 kb–1 kb marker (GeneDirex), W1 and W2 – untransformed control, 1-20 are putative fungal transformants. (B) Amplification of the rec8 ORF using P11 and P13 primers, true mutants do not show amplification. 1 kb–1 kb marker (GeneDirex), W1 and W2 – untransformed control, 1-20 are putative fungal transformants. Transformants 1 and 12 are true deleted strains for rec8 locus. [file Image_4.JPEG]

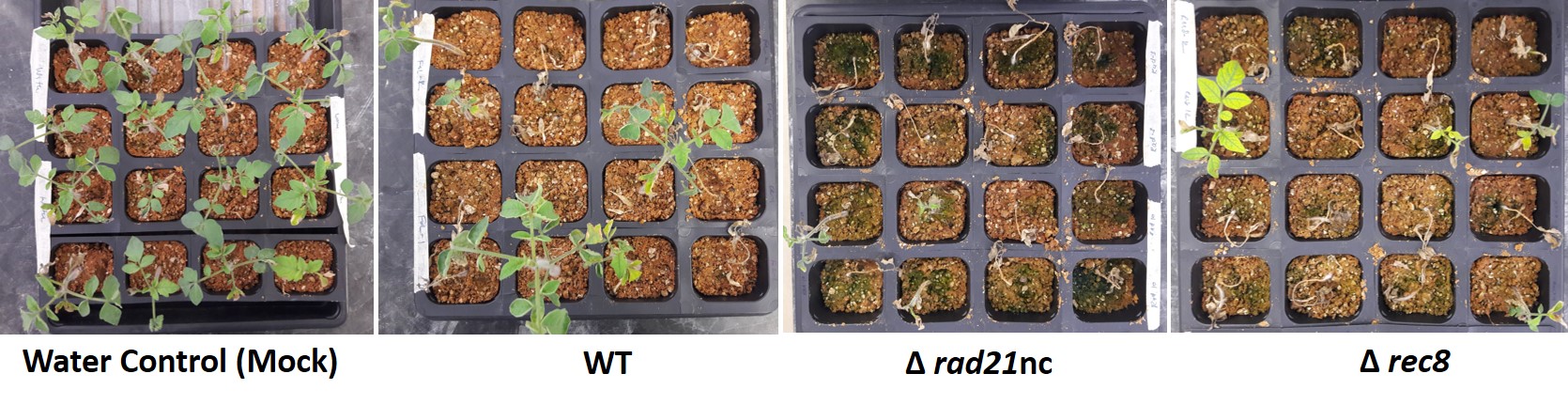

Supplement: FIGURE S5 — Tomato wilt disease caused by F. oxysporum strains mutated in rec8 or rad21nc. Tomato plants infected with WT, Δrad21nc, or Δrec8 fungal strains and water control (mock) after 21 dpi under controlled growth condition as described under the Section “Materials and Methods.” [file Image_5.JPEG]
