# Supplementary material for: Alternative Functional rad21 Paralogs in Fusarium oxysporum
Source: Front Microbiol. 2019 Jun 18;10:1370. doi: 10.3389/fmicb.2019.01370 (PMC6591460; doi:10.3389/fmicb.2019.01370)
Supplement: TABLE S1 — 90 DNA Repair proteins that were used to build the species tree that is presented in Figure 1. [file Table_1.DOCX]

**Table S1.** **Primers used in the study.**

| Primer Name | Gene name and Purpose | Sequences (Complimentary overhangs were underlined) |
| --- | --- | --- |
|  |  | **For Rad21nc deletion** |
| P1 | Rad21nc-F1 | CCCTCGGAATTACACAGGAATTA |
| P2 | Rad21nc-F2 | GCTCCTTCAATATCAGTTAACGTCGCACAGCAATAAGCACAGTAAG |
| P3 | Rad21nc-F3 | GACTATGAAAATTCCGTCACCATGGAGATGAGCCAAGTGATATG |
| P4 | Rad21nc-F4 | TTCCTCCCTATTAATAAAGACCTTAGAG |
| P5 | Rad21nc-F’HYG | CTTACTGTGCTTATTGCTGTGCGACGTTAACTGATATTGAAGGAGC |
| P6 | Rad21nc-R’HYG | CATATCACTTGGCTCATCTCCATGGTGACGGAATTTTCATAGTC |
| P7 | Rad21nc-NestF1 | GATTGGGCAAGGTAATGC |
| P8 | RP_HY | CTCTGATAGAGTTGGTCAAGACC |
| P9 | FP_YG | GAGCCTGACCTATTGCATCTC |
| P10 | Rad21nc-NestF2 | ACTTTAGGGCTATAAGGCAGACC |
| P11 | Seq_Rad21nc_FP | ACTCCAAACCTTCTCCCCTC |
| P12 | Seq_Rad21nc_RP | GTGGTCTTTGTTTCGTCCTG |
| P13 | ORF_Rad21nc_RP | GAACAACACCAAGTAGTAACTGACTG |
|  |  | **For Rec8 deletion** |
| P1 | Rec8-F1 | GAGTGCTGATAGCCAATAAACTG |
| P2 | Rec8-F2 | GCTCCTTCAATATCAGTTAACGTCTATAGAACATGCTGCTCCCTG |
| P3 | Rec8-F3 | GACTATGAAAATTCCGTCACCAGCGATGACTTTACGATCTCA |
| P4 | Rec8-F4 | GAATTCCATACGAGTTTCCAGTCTAT |
| P5 | Rec8-F’HYG | CAGGGAGCAGCATGTTCTATAGACGTTAACTGATATTGAAGGAGC |
| P6 | Rec8-R’HYG | TGAGATCGTAAAGTCATCGCTGGTGACGGAATTTTCATAGTC |
| P7 | Rec8-Nest-F1 | ACCTCGAGATCGTCGTACC |
| P8 | RP_HY | CTCTGATAGAGTTGGTCAAGACC |
| P9 | FP_YG | GAGCCTGACCTATTGCATCTC |
| P10 | Rec8-Nest-F2 | ATCTCTGACAACCTGATGCAC |
| P11 | Seq_Rec8_FP | AAGCTTTCAACCTCACCTACTAACT |
| P12 | Seq_Rec8_RP | CCACATATGCTGTCAATACCTATG |
| P13 | ORF_Rec8_RP | TCAGCGTCTGAGAGCATATAAC |
|  |  | **For HYG cassette amplification** |
| P14 | FP_Ptrpc_HYG | GACGTTAACTGATATTGAAGGAGC |
| P15 | RP_ Ttrpc _HYG | TGGTGACGGAATTTTCATAGTC |
|  |  | **For RT-PCR for rad21 paralogs in *Fusarium oxysporum* f. sp. *lycopersici*** |
| P16 | rad21c_F | CTGACGATGTCTCCCAAGTACCT |
| P17 | rad21c_R | CCCGGTCTTCGTCGATGT |
| P18 | rad21nc_F | CTCCCTCGGGACCCTTTCT |
| P19 | rad21nc_R | GGAGCTTCGTCCTTCCATCA |
| P20 | rec8_F | GGACATGGTAGCGCACAGAAA |
| P21 | rec8_R | GATCACTCCAGCGCTCAATTG |
| P22 | act1_F | GAGGGACCGCTCTCGTCGT |
| P23 | act1_R | GGAGATCCAGACTGCCGCTCAG |
